# Field-deployable lightweight YOLOv8n for real-time detection and counting of Maize seedlings using UAV RGB imagery

**DOI:** 10.3389/fpls.2025.1639533

**Published:** 2025-09-08

**Authors:** Pengbo Feng, Zhigang Nie, Guang Li

**Affiliations:** ^1^ College of Information Science and Technology, Gansu Agricultural University, Lanzhou, China; ^2^ State Key Laboratory of Aridland Crop Science, Gansu Agricultural University, Lanzhou, Gansu, China; ^3^ Hexi University, Zhangye, Gansu, China

**Keywords:** Maize seedling, small target, lightweight, YOLOv8n, Grad-CAM++

## Abstract

The aim of this study is to propose a lightweight YOLOv8n maize seedling detection algorithm that incorporates multi-scale features to address the problems of large number of model parameters and computation, low real-time performance, and small detection range of the existing maize seedling detection models during plant detection. By fusing RepConv with HGNetV2 using the idea of reparameterisation, a Rep_HGBlock structure is designed to form a new lightweight backbone network, Rep_HGNetV2,; BiFPN is introduced into the neck network portion of the model to enhance the interactive fusion of bidirectional information flow between multiple scales and hierarchies; and a fusion task decomposition, dynamic convolutional alignment is designed, DFL (Distribution Focal Loss) ideas, TDADH, a task dynamically aligned detection head, which uses shared convolution and dynamically aligns the tasks of classification and localization to extract features; and Grad-CAM++ technique is used to generate a heat map for model detection, visualize effective features of the target and understand the model focus region. The experimental results show that the improved model achieves a detection accuracy of 96.5%, which is basically the same as the original model. The weight size, number of parameters, and computational FLOPs are reduced to 3.5 MB, 1.58 M, and 7.4 G, respectively, which are reduced by about 43%, 47%, and 8.6%. The frame rate FPS is only reduced from 149.98 to 146.3, a reduction of about 2.4%. The results show that the lightweight model has high recognition accuracy, speed and low complexity, which is more suitable for practical deployment in resource-constrained edge devices, UAVs, and embedded systems, and is able to provide technical support for the precise management of maize during the seedling stage of drip irrigation water-fertilizer integration.

## 1 Introduction

Maize is the second largest grain crop in China, with an overall trend of steady growth in acreage and production, and is widely used in food, feed, biodiesel and industry, which is of great significance to China’s production development. The Hexi Corridor in Gansu, as an important production area in the main irrigated maize production region in Northwest China, securing production is a prerequisite basis for improving yield. Plant density at the seedling stage plays a key role in detecting maize germplasm quality, early breeding decisions, improving variety emergence, timely replanting, and key information on traits related to crop quality and yield. It can also be used as a parameter to understand the impact of different field crop management activities on crop yield. Farmers can use this information to adopt necessary agricultural practices to compensate for deficiencies in the current growth stage of the crop (Shirzadifar et al., 2020). Accurate and rapid detection and calculation of maize seedling emergence number is an important guarantee for increasing maize yield and biomass, and an important prerequisite for carrying out precision agriculture techniques such as automated precision weeding, smart irrigation, and pest and disease detection.

The traditional estimation of seedling number in the field is determined by manually calculating the number of plants in selected plots in the field and visually assessing seedling growth. The manual survey method is costly, labor intensive, time consuming and inefficient (Jiang et al., 2016). This method is also prone to human error, resulting in insufficient or inaccurate planting information (Peng et al., 2022). The rapid development of UAV technology has effectively bridged the gap between ground-based sensing and high-altitude remote sensing, enabling more flexible and precise field-level crop monitoring (Roth et al., 2020).Compared with traditional ground surveys and satellite imaging, UAVs offer significant advantages in terms of resolution, timeliness, cost-effectiveness, and operational flexibility, making them especially suitable for small- to medium-scale farmland management and high-frequency crop status assessment. UAVs carry a variety of sensors such as RGB, multispectral, hyperspectral, and thermal imaging to collect field data using a variety of methods to estimate different field information such as yield (Dhaliwal and Williams, 2024; Li et al., 2020; Tanaka et al., 2021), biomass (Shu et al., 2023; Yu et al., 2023), LAI (Castro-Valdecantos et al., 2022; Liu et al., 2021), crop height (Chang et al., 2017), and temperature (Zhang Z. et al., 2021).

Current research on the number of crop emergence mainly focuses on the use of computer vision and machine learning(ML) methods. In the early stage, UAVs were used to obtain field vegetation images to segment crops and vegetation by combining color features and morphological features, and to predict the number of crop plants to construct a vegetation distribution model. Liu et al. compared different color spaces to select a color model for segmentation of UAV images, used morphological processes to extract corn seedling morphology, and compared the recognition results of different detection algorithms to extract information on the number of corn plants (Liu et al., 2022). Such methods are susceptible to factors such as terrain, data collection height, and selected crop area, and cannot learn information such as crop size and texture, and the recognition effect is difficult to guarantee. At present, deep learning(DL) methods are gradually replacing traditional machine vision methods due to their strong detection accuracy and generalization ability, and are widely used in the detection and counting of animals (Weng et al., 2024), the detection of picking points or key points (Yang et al., 2024), the detection of pest and disease identification (Ahmad et al., 2022; Dong et al., 2024; Jia et al., 2023), and the segmentation of values (Liu et al., 2024). The research in crop counting covers a wide range of crops such as tea (Li et al., 2023; Xia et al., 2024), watermelon (Jiang et al., 2024), wheat (Li et al., 2024), apple (Tian et al., 2019), grape bunches (Li et al., 2021), chilli (Ma et al., 2024), longan (Li et al., 2021), oilseed rape (Su et al., 2023)and so on. Jin et al. collected very low altitude high-resolution images with different sowing dates, densities, genotypes, flight heights and growth stages at different experimental locations to separate background and green pixels to identify and extract crop rows to estimate the number of plants contained in the images to estimate the plant density of wheat at the seedling stage. The experimental results showed that the RMSE and relative RMSE of the method were 34.05 plants/m^2^ and 14.31% for 270 samples at three sites with a deviation of 9.01 plants/m^2^ (Jin et al., 2017).A new method for counting, locating and grading rice plants was proposed by Bai et al. The method consists of a feature extractor front-end and three feature decoder modules consisting of a density map estimator, a plant location detector and a plant size estimator. The rice plant attention mechanism and positive and negative losses are designed to improve the ability to distinguish plants from background as well as to estimate the quality of the density map, and a UAV rice counting dataset containing 355 images and 257,793 manually labelled points is built. The experimental results show that the mean absolute error and root mean square error of the proposed RiceNet are 8.6 and 11.2, respectively (Bai et al., 2023).Osco et al. proposed a novel DLmethod based on Convolutional Neural Networks (CNNs) capable of simultaneously detecting planting rows and counting plants from UAV images, which performs well on cornfield and citrus orchard datasets that Accurate counting and localization of high-density planting configurations for different types of crops was achieved. The detection accuracy in the corn dataset is 0.856, which performs well and outperforms the results of other deep networks on the same task and dataset (Osco et al., 2021). Li et al. collected data of maize at the V6 stage using drones and combined the YOLOv5 detection model with the Kalman filter algorithm to achieve tracking and counting of maize plants. The average precision of the detection model on the test dataset, mAP@0.5, was 90.66% (Li et al., 2022).LU et al. used UAV RGB images and DLalgorithms to apply a semi-automatic labelling method based on Segment Anything Model (SAM) to achieve accurate identification of maize seedlings under weed interference. The experimental results show that the multi-category average precision (mAP) for detecting corn seedlings is 94.5% and 88.2% at 15 m and 30 m flight altitude, respectively (Lu et al., 2024). Zhang et al. proposed a seedling acquisition and detection model (FE-YOLO) based on a feature enhancement mechanism, which achieves fast acquisition of maize seedling plants based on their target size and spatial texture features. Experiments showed that the model’s mAP and recall reached 87.22% and 91.54%, respectively (Zhang H. et al., 2021). With the rapid development of precision agriculture, the practical application scenarios of crop monitoring are continuously expanding, particularly on resource-constrained platforms such as unmanned aerial vehicles (UAVs), embedded systems, and Internet of Things (IoT) devices. In the early growth stage of maize (e.g., two-leaf and one-heart stage under integrated water-fertilizer irrigation), seedlings are typically small, densely planted, and visually similar to the background soil. These factors pose significant challenges for accurate target detection in real-world field conditions, including low target visibility, high false detection rates, and deployment difficulties. Although existing object detection models have achieved high accuracy, they often rely on large model sizes, high parameter counts, and significant computational costs. These characteristics hinder their real-time applicability and deployment on edge devices with limited processing power and memory.

To address these challenges, this study proposes a novel lightweight target detection model, YOLOv8-FLY, specifically designed for real-time monitoring of maize seedlings in actual farmland environments. The model integrates RepConv with the HGBlock structure from HGNetV2 to form a new lightweight multi-scale backbone module, Rep_HGBlock, which enhances feature representation for small targets while reducing network complexity. A Bidirectional Feature Pyramid Network (BiFPN) is introduced in the neck layer to improve multi-scale information fusion and reduce computational burden. Furthermore, we design a lightweight detection head, TDADH, based on GroupNorm, shared convolution, and task-decoupled interaction mechanisms to enhance detection capability with fewer parameters, facilitating deployment on edge devices.

To validate the proposed model in real-world agricultural scenarios, we constructed a maize seedling dataset using UAV-acquired RGB imagery from 3-meter altitude in a drip-irrigated field. We further developed a practical detection system capable of processing both image and video streams, displaying real-time plant positions, target counts, and frame rates, and supporting live UAV camera input. The proposed model achieves comparable accuracy (96.5% mAP) to the original YOLOv8n while significantly reducing weight size (3.5 MB), parameter count (1.58 M), and computational cost (7.4 GFLOPs), with only a 2.4% reduction in frame rate. The YOLOv8-FLY model and the supporting monitoring system demonstrate strong potential for real-time, efficient, and scalable deployment in actual maize field environments, providing timely support for early-stage crop management and precision agriculture applications.

## 2 Materials and methods

### 2.1 Collection and production of maize seedling data set

**Figure 1:**
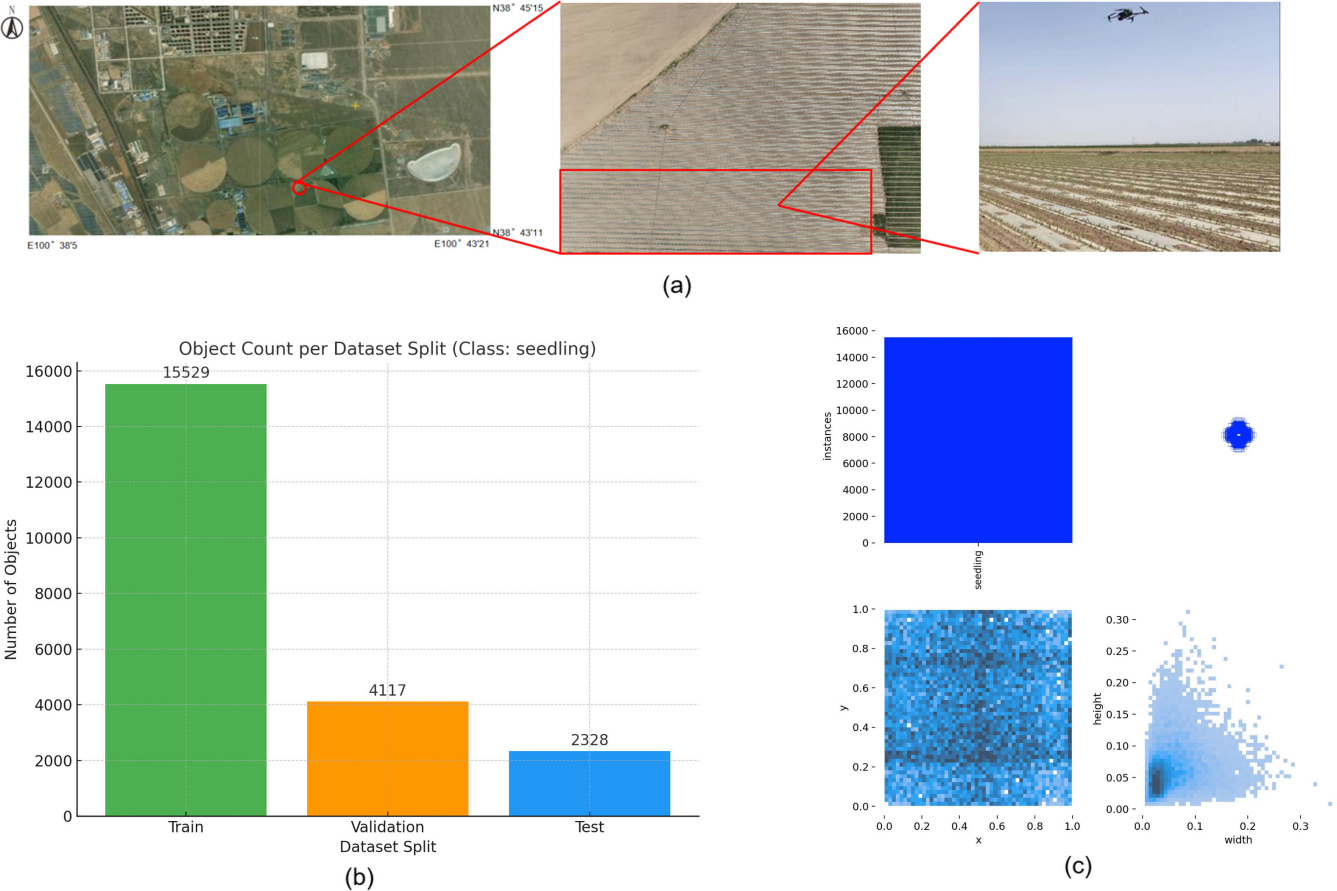
Experimental Site and Distribution of Dataset Proportion and Size. **(a)** Experimental Site, **(b)** Proportion of Dataset Instances, **(c)** Size and Distribution of Training Set Labels.

A maize seedling dataset was constructed in this study. The experimental site is shown in **Figure 1a**, located in Huarui Ranch, Minle County, Gansu Province, China (latitude 38°44′3.32″N, longitude 100°42′5.03″E; altitude 1,683 m above sea level). The climate of this area belongs to the temperate continental arid steppe climate type, with low precipitation, arid and sandy climate, and the soil of farming is dominated by scrub desert soil. The corn planting method is integrated mechanical drip irrigation with film cover, and the irrigation method is drip irrigation mode based on water-fertilizer integration technology. Drip irrigation pipes were laid in the middle of narrow rows with a width of 25 cm, and the spacing between adjacent wide rows was 50 cm. The short growth period of maize seedlings and the size of the leaf area of the plants during the growth period would affect the detection accuracy of maize plants, and in order to dynamically monitor the emergence of maize plants and detect the quality of planting in a timely manner to adopt a reasonable field management strategy, We selected 2-leaf and 1-center seedlings approximately 20 days after sowing from the flooded field for this experiment. In this experiment, a DJI Mavic Classic 3 (Shenzhen DJI Innovative Technology Co., Ltd.) drone equipped with an RGB camera was selected, and a total of 1,213 images with a resolution of 5280 × 2970 pixels were collected and stored in JPG format using the drone to shoot at the test site. The drone was flown at an altitude of 3 m above the ground to ensure high-resolution image capture suitable for small-target detection, and the drone lens was flown at a constant speed of 1 m/s vertically on the ground during the shooting, and the flight path was manually operated. Based on the flight altitude and camera specifications, the ground sampling distance (GSD) of the collected images was approximately 0.07 cm/pixel. This ultra-fine resolution ensured that subtle morphological features of maize seedlings were preserved, which is critical for accurate detection of small and densely distributed plants in early growth stages. The low-altitude setting was chosen after initial trials at 20 m and 50 m revealed substantial loss of detail and increased shadow interference, which negatively affected model training and detection robustness. The data were selected on 3 and 4 May 2024, with the time period of 9:00-14:00 sunny, less cloudy and windless weather, to reduce the impact of light refraction and wind and sand on the flight range of the UAV and the quality of data collection, and to improve the efficiency of data collection. The data collection process over the experimental plot took approximately 45–60 minutes.

**Figure 2:**
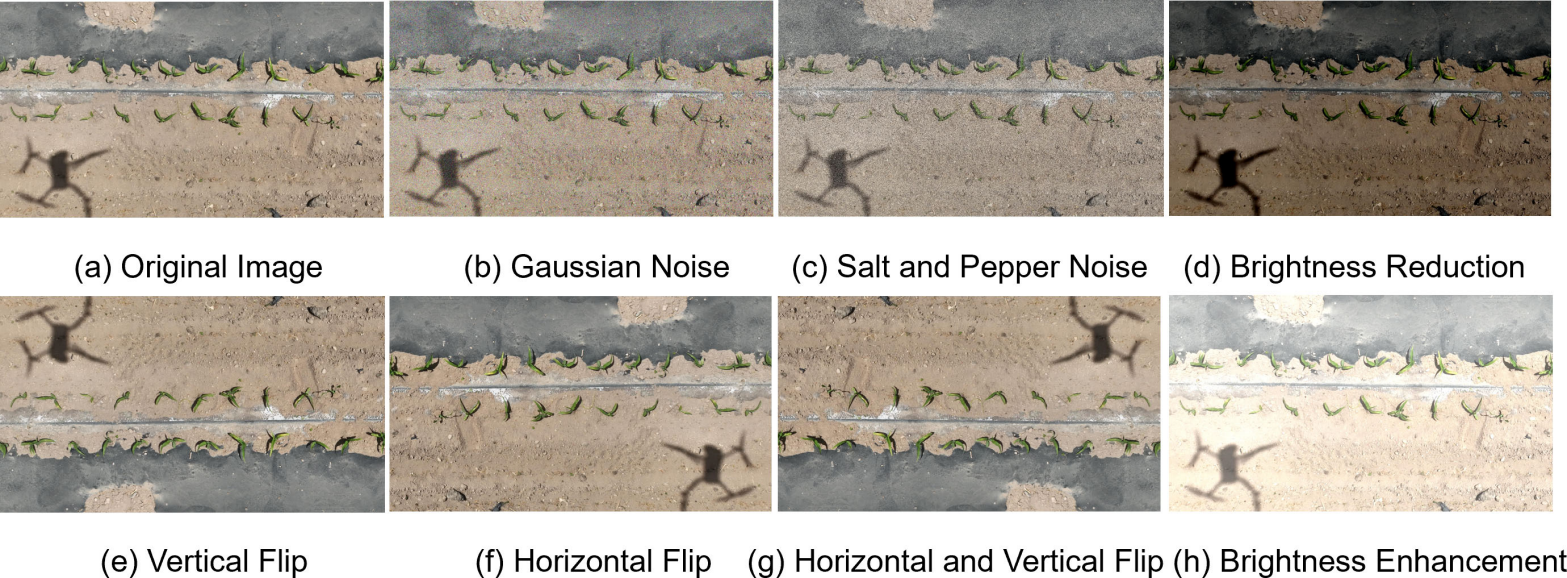
Examples of Dataset Augmentation. **(a)** Original Image, **(b)** Gaussian Noise, **(c)** Salt and Pepper Noise, **(d)** Brightness Reduction, **(e)** Vertical Flip, **(f)** Horizontal Flip, **(g)** Horizontal and Vertical Flip, **(h)** Brightness Enhancement.

A total of 21,974 labeled instances were manually annotated using the open-source software LabelImg 1.8.6 (Tzutalin, 2015). The annotation followed the YOLO format, with each.txt file containing the instance class, normalized coordinates of the bounding box (center x, center y, width, height), and the number of instances per image. The dataset is divided according to the ratio of training set: validation set: test set as 7:2:1 respectively, and the ratio of the number of instances labelled in the respective instances is 15529:4117:2328, as shown in **Figure 1b**. The dataset was enhanced by horizontal flip, vertical flip, 90-degree rotation, adding noise, and brightness adjustment enhancement in one or a combination of several ways, and a total of 65,600 images and labelled files were generated after enhancement. Some of the enhanced data are shown in **Figure 2**. To avoid over-enhancement of the same image in the dataset, leading to sample duplication, we randomly selected one image from all images (original image + enhanced image) generated after enhancing each original image in the dataset as the final sample. This means that for each original image ID, only one representative image—either the original or one randomly selected augmented version—was retained. This ensures that each real-world sample appears only once in the final dataset, while preserving diversity in lighting, orientation, and image quality. Such a strategy avoids synthetic redundancy, reduces the risk of overfitting, and better simulates the variations encountered in actual UAV field imagery. In order to ensure the quality of the dataset, the collected data are screened, and the duplicate images and lower quality graphics are removed from the images and 993 high quality images are finally selected as the original dataset. The size and distribution of instance labels in the training set are shown in **Figure 1c**. The distribution of target sizes is shown in the two - dimensional heatmap histogram in the lower - right corner of **Figure 1c**, where a deeper blue indicates a higher number of targets that appear in the corresponding width - height combination, namely, that the density is higher. The figure shows that the relative widths of the majority of the targets are concentrated in the range of 0.02 to 0.07 and the heights in the range of 0.02 to 0.10. In the original image with a resolution of 5280 ×2970, the real - life dimensions correspond to approximately 106 to 370 pixels (width) and 59 to 297 pixels (height). This indicates that the targets in this data set are densely distributed in space and relatively small in size, representing a typical small - object detection task.

### 2.2 Improved YOLOv8-FLY network structure

**Figure 3:**
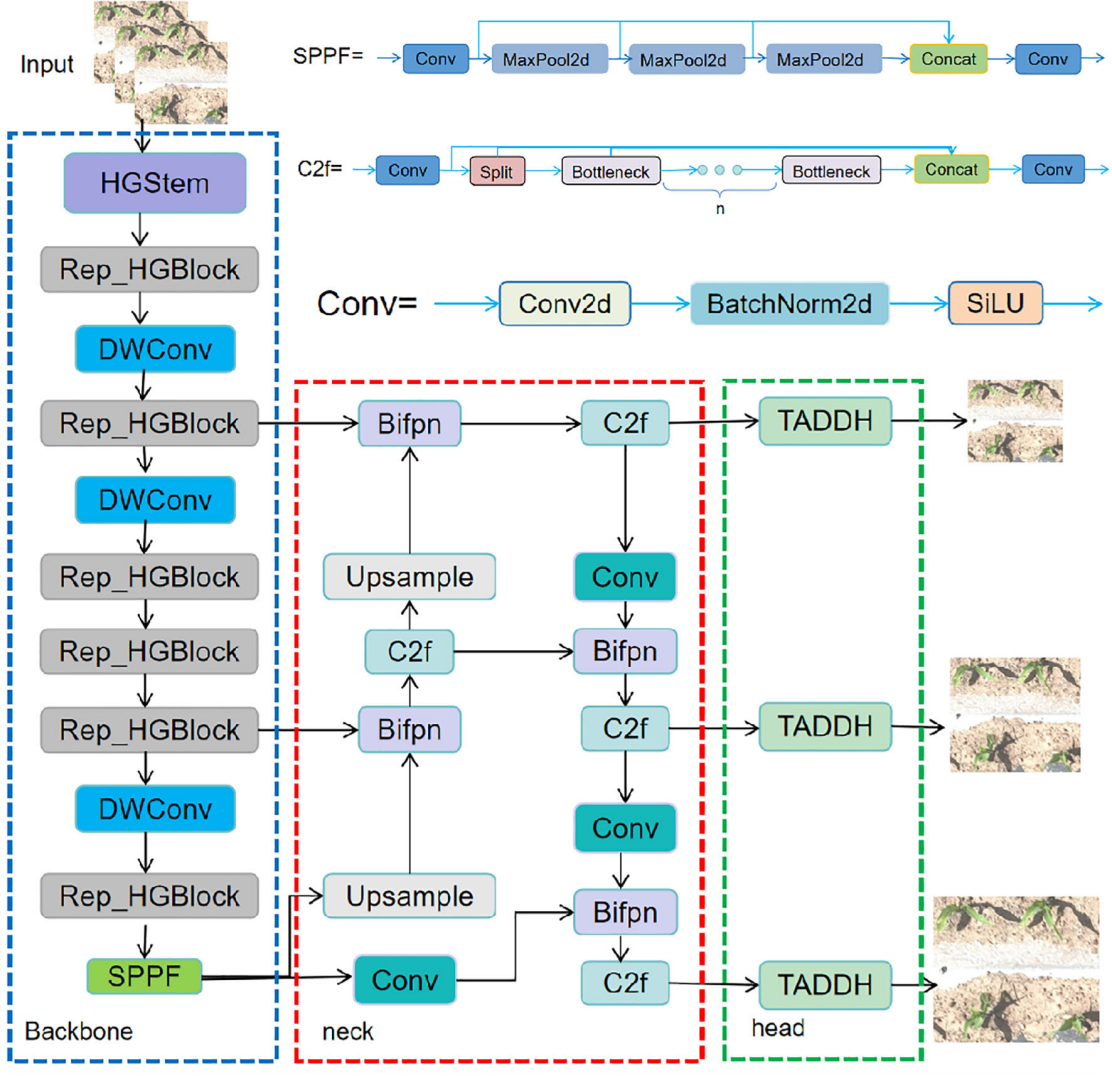
Structure of the Improved YOLOv8-FLY.

In order to solve the problems of small size, irregular posture, high density and limited computational resources of deployed devices of targets in the field of maize seedlings, a lightweight target detection model YOLOv8-FLY is designed in this study. The structure of the model is shown in **Figure 3**, which consists of the following three parts of the core improvement:

Firstly, in the Backbone part, a lightweight structural module, Rep_HGBlock, is designed. First, in the Backbone part, a lightweight structural module, Rep_HGBlock, is designed, which combines the structural reparameterisation feature of RepConv with the multi-scale local feature extraction capability of HGBlock in HGNetV2. This module can effectively enhance the representation of small targets while maintaining high accuracy, and significantly reduce the number of model parameters and computation to improve the recognition of dense seedlings in the field.

Secondly, a BiFPN is introduced into the Neck layer, which enhances the information flow interaction between multi-scale features, improves the model’s ability to perceive targets at different scales, and at the same time, reduces the parameter redundancy and computational overhead of the layer, so as to effectively adapt to the problem of image resolution difference in practical applications.

Finally, the lightweight detection head TDADH is designed by combining GroupNorm with shared convolution and task interaction mechanism, which reduces the number of detection head parameters on the basis of guaranteeing the detection accuracy, improves the inference efficiency, and is more suitable for deploying in real-time monitoring platforms such as agricultural drones and edge devices.

In summary, the YOLOv8-FLY model takes into full consideration the multiple requirements of detection accuracy, computing efficiency and resource consumption in actual field applications in its structural design, and achieves the overall lightweight and deployment friendliness of the model while maintaining accuracy, which has good potential for practical applications.

### 2.3 Rep_HGNetV2 lightweight network

In the images of maize seedlings captured by field UAVs, the targets generally present features such as small size, high density, and weak texture information, coupled with the fact that the system needs to be deployed in resource-constrained edge devices, thus placing higher demands on the model’s ability to recognize small targets and the degree of lightweighting. In order to balance the detection accuracy and model efficiency, a lightweight multi-scale backbone network, Rep_HGNetV2, is designed based on the HGNetV2 network architecture, which is used to enhance the feature extraction capability and reduce the complexity of the model for maize seedling small targets.

The HGNetV2 network architecture has excellent hierarchical semantic extraction capability and lightweight characteristics, which can effectively improve the detection performance of the model in complex backgrounds. However, in order to further reduce the number of parameters and improve the efficient inference ability, this study introduces the idea of structural reparameterisation on the basis of HGNetV2, fuses RepConv and HGBlock, and proposes a new lightweight module, Rep_HGBlock, which is used to construct the backbone network of the YOLOv8-FLY model.

**Figure 4:**
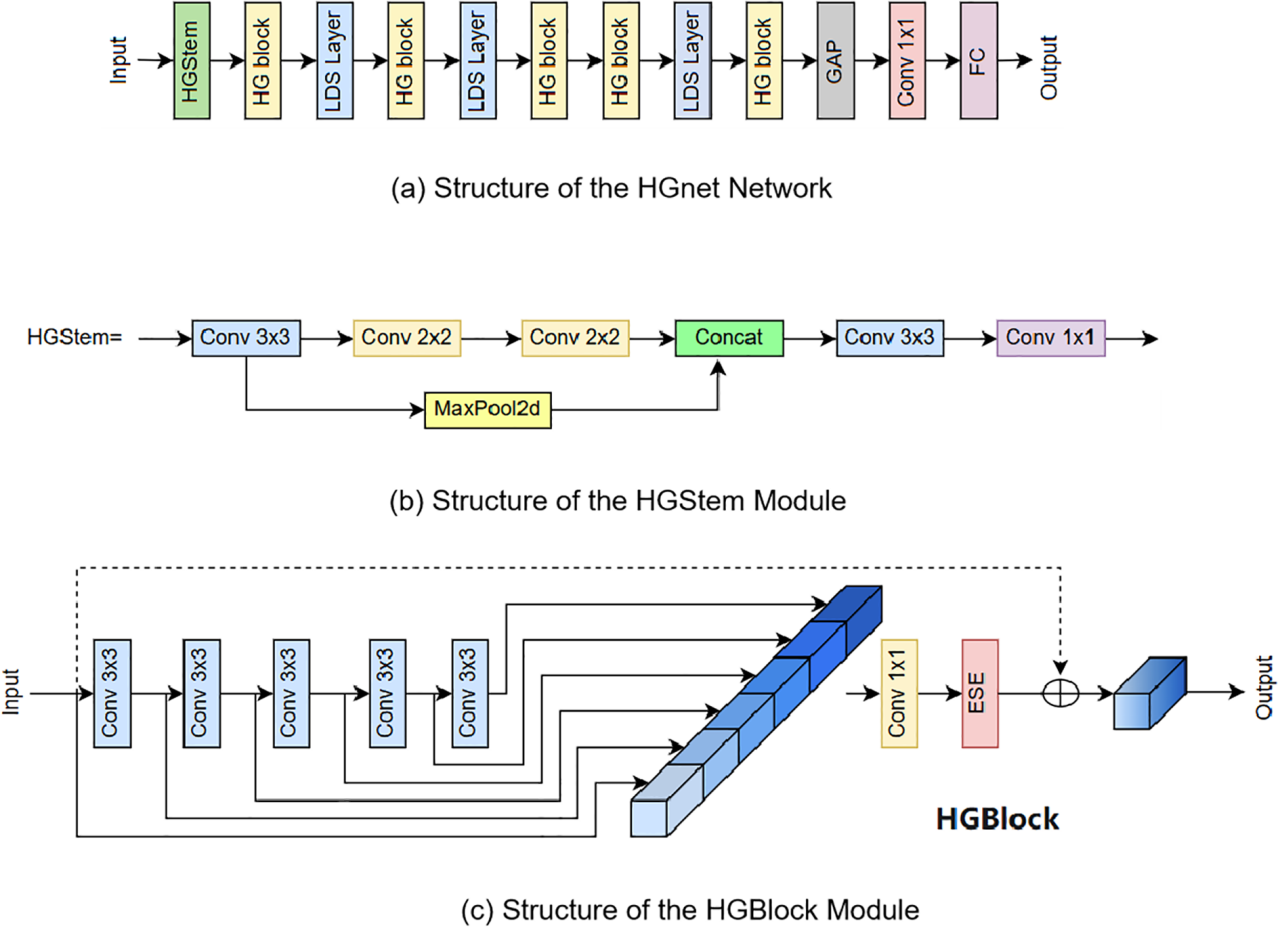
Structure of HGnet and Its Internal Modules. **(a)** Structure of the HGnet Network, **(b)** Structure of the HGStem Module, **(c)** Structure of the HGBlock Module.

The overall structure of HGNet consists of HGStem, four HG Stages, Global Average Pooling (GAP), Convolution and Fully Connected (FC) layers. The HGStem class implements the StemBlock of PHGNet and contains multiple convolutional layers and a maximum pooling layer. The lightweight convolutional operations are designed to extract image features quickly and efficiently in the early stages of the network and reduce the number of parameters and computational complexity of the model by reducing the spatial resolution of the feature map. By using different sizes of convolutional kernels and pooling layers, HGStem is able to capture features at different scales to provide useful feature representations for the deeper layers of the network, which helps the network learn richer visual information and enhances the network’s adaptability to targets of different sizes. Each HG Stage contains a large number of standard convolutional HG Blocks, and the HG_Block consists of multiple ConvBNAct and ESE modules. The design of the HGBlock aims to improve the performance and generalization of the network through lightweight convolutional operations and effective feature fusion strategies. The structure of the HGnet network and the structure of the HGStem, HGBlock modules are shown in **Figure 4**.

HGNetv2 (Zhao et al., 2024)proposed by Baidu Paddle-Paddle team keeps the most basic constituent unit of HGNet, ConvBNAct, unchanged, and adds the branch residual use_lab structure in the activation function part, which controls the shunt size by scale. Use LightConvBNAct module to replace the ESE module in HGNet. In HGNetv2 a large number of stacked convolutional modules are used to process the data hierarchically to extract information from each layer. The architecture utilizes a hierarchical approach to feature extraction, where complex patterns can learn target features at different scales and levels, improving the network’s ability to process complex image data. This hierarchical and efficient processing is essential for image classification tasks to accurately predict complex patterns and features at different scales.

RepConv is derived from the VGG (Ding et al., 2021) network and its core lies in reparameterisation. The main idea of reparameterisation is to fuse convolution (Conv) and normalization (BN) to reduce the amount of operations to stabilize the training process. RepConv is a fusion of 3x3 convolution and BN to unify different sizes of convolution as 3x3.RepConv is mainly used to improve the inference speed while keeping the accuracy not degraded. RepConv uses a different structures in the training and inference phases with the aim of greatly reducing the complexity of the inference phase. In the training phase, a multi-branch structure is used for training to learn rich feature information of the target, but in the inference phase, a single branch is used, which retains the accuracy of multi-branch in training and the speed of single branch in inference. backbone uses RepBlock modules in the training phase, but in the inference phase, these RepBlock modules are replaced with 3x3 convolutional blocks with ReLU activation function. of 3x3 convolutional blocks.

The reparameterisation process is different for different branches: the 3x3 Conv is directly fused with the BN layer; the 1x1 Conv is first padding into a 3x3 Conv and then fused with the BN layer; the identity is converted into a 1x1 Conv and then into a 3x3 Conv, and finally fused with the BN layer. The RepConv is a 3x3 convolution and ReLU activation function, compared to the ordinary convolution block, less of which is the BN layer, the core idea is that Conv2D is fused with DB phase equivalent to a 3x3 convolution. The formula for the fusion of convolution Conv2D and batch normalized BN is as follows:


(1) Conv(x)=W(x)+b

(2) $$BN\left(x\right)=\gamma \cdot \frac{\left(x-mean\right)}{\sqrt{\mathrm{var}}}+\beta$$

Where, Equation 1 is the convolutional layer formula represents the standard convolutional operation, W is the convolutional kernel and b is the bias term.

Equation 2 is the BN layer formula, which is the standard form of Batch Normalization. γand βare the learnable parameter weight and bias values of the BN layer. According to the process of convolution block, first through the convolution layer, and then the BN layer, the convolution result of Equation 1 can be written into the following form by substituting the formula in Equation 2, as shown in Equation 3:


(3) $$BN\left(C\text{onv}\left(x\right)\right)=\frac{\gamma }{\sqrt{\mathrm{var}}}\cdot W\left(x\right)+\left(\frac{\gamma \cdot \left(b-mean\right)}{\sqrt{\mathrm{var}}}+\beta \right)$$

Where, $\frac{\gamma \cdot W\left(x\right)}{\sqrt{\mathrm{var}}}$ is the weight after fusion of BN layer and $\frac{\gamma \times \left(\text{b}-\text{mean}\right)}{\sqrt{\text{var}}}+\beta$ is the bias after fusion of BN by convolutional layer.

**Figure 5:**
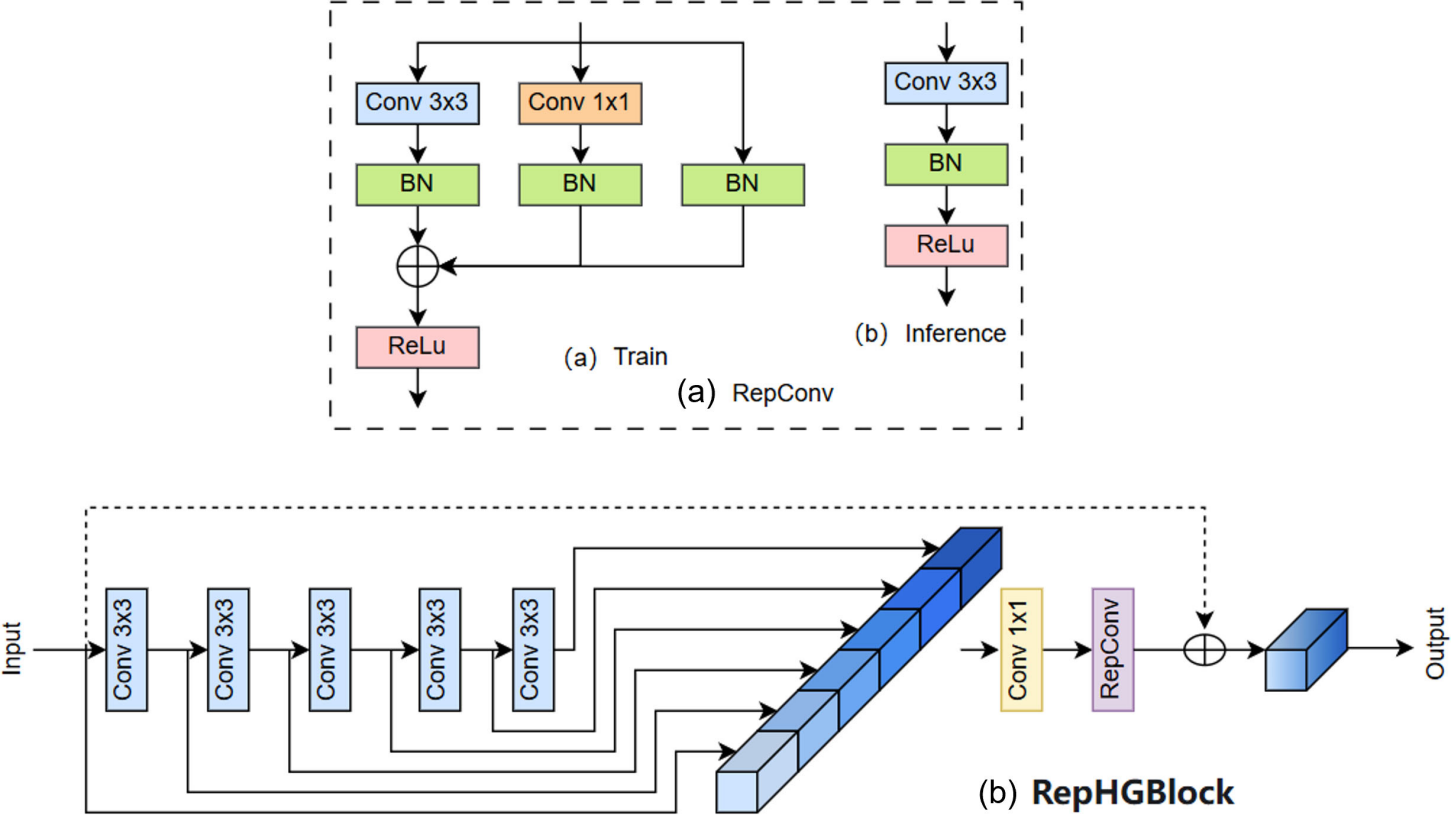
**(a)** Structure of the RepConv Module, **(b)** Structure of the Rep_HGBlock Module.

A new backbone network Rep_HGNetV2 consisting of HGStem, Rep_HGBlock and DWConv is designed by using the idea of reparameterisation in the improved algorithm. The Rep_HGBlock structure is designed by fusing RepConv with HGBlock in HGNetV2, which ensures the detection speed and algorithmic performance while reducing the structure of Rep_HGBlock is shown in **Figure 5b**, which ensures the detection speed and algorithmic performance while reducing the number of parameters and computation amount of the model to achieve real-time maize seedling plant detection.

### 2.4 BiFPN module

Due to the small maize seedling plants to be detected in this study, there are significant differences in the morphology of different plants and the influence of complex environments such as mutual shading between multiple plants, which increases the complexity of the seedling plant identification and detection task. Therefore, the model must have a strong multi-scale feature fusion capability. The Neck part of YOLOv8n contains a PANet structure, which extracts features at different scales through the Backbone for fusion. The PANet is based on the FPN and adds extra bottom-up paths to achieve feature fusion, which effectively reduces the loss of feature information transmission and enhances the model’s ability to detect multi-scale targets. targets. However, while the PANet structure enhances the performance of target detection, its feature information fusion ability is still limited and the computational efficiency needs to be improved. Therefore, there is still room for optimization in detecting small targets and targets in complex environments.

**Figure 6:**
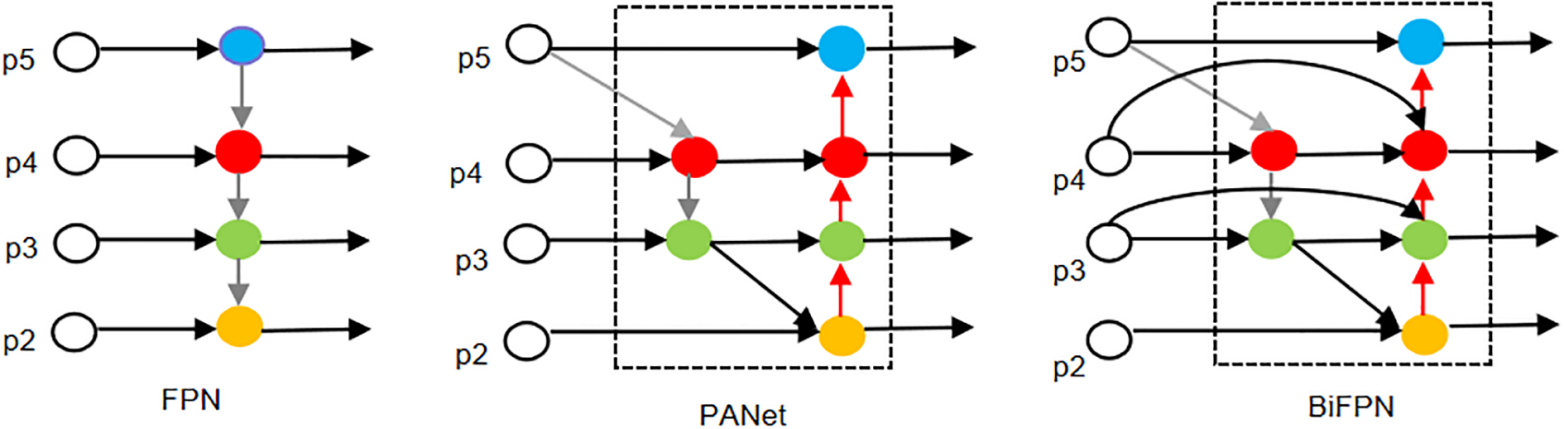
Structures of FPN, PANet, and BiFPN.

In order to overcome the limitations of the PANet structure, this study introduces the BiFPN (Tan et al., 2020) structure in the neck part of the YOLOv8n model. BiFPN has an efficient multi-scale feature fusion capability with fewer parameters and computational complexity, which is suitable for embedded devices and lightweight deployments; it is based on the idea of the PANet to optimize the feature fusion process using the adaptive feature tuning mechanism, and introduces learnable learning weights to learn the importance of different input features to adjust the features of different layers to better match the needs of different tasks. Additional lateral jump connection paths are added between the original input and output nodes at the same level, and the features extracted by the backbone network are integrated into the feature map to be detected, so that the network integrates more feature information into the feature map to be detected without significantly increasing the computational cost, which effectively improves the problem of the loss of feature information in the image due to the change of the resolution of the feature map. Small targets are more likely to be ignored or misjudged because they occupy fewer pixels in the image. The improved Neck network can fuse more target location and detail information, and improve the contextual understanding of the target seedling, so that the network better focuses on the area where the small target is located, more keenly detects and locates the small target of the maize seedling to reduce the false alarms or omissions, and improves the accuracy and overall performance of the detection of the small target. The structure of the BiFPN is shown in **Figure 6**.

**Figure 6** Note: The grey arrow part is the top-down pathway, which conveys the semantic information of the high-level features; the red arrow part is the bottom-up pathway, which conveys the location information of the low-level features; and the black arc part is the cross-scale connection, which is weighted fusion and bi-directional cross-scale connection by adding a residual jump connection and a bi-directional pathway to achieve the weighted fusion and the bi-directional cross-scale to connection.

### 2.5 TDADH detection head

As this study is an algorithm for real-time field detection and edge deployment of maize seedlings, the detection task is usually carried out in field environments, so the lower computational complexity and lighter weight model facilitates practical deployment on resource-constrained devices such as UAVs. In the YOLOv8n model, the number of parameters and computation of the detection head account for a high proportion of the model, so this study improves the detection head to ensure the detection accuracy while reducing the complexity of the model and realizing the model’s lightness. The detection head of the YOLOv8n model adopts a decoupled head, and each detector head has two branches, and each branch contains two 3×3 convolutions and one 1×1 convolution. The reused convolutions in the detection head lead to a significant increase in the model computation and number of parameters. At the same time YOLOv8n adopts the allocation strategy of tal in the target detection process classification branch and localization branch are independent of each other the lack of interaction between the two tasks, there is a problem of inconsistency between the prediction of the localization and classification branches leading to inaccurate prediction of the location and reduced prediction accuracy. In order to solve this problem, this paper designs a TDADH detection head, whose core idea is to change the original two branches to one branch, and extract features by using shared convolution and dynamically aligning the tasks of classification and localization. The detection head incorporates the ideas of task decomposition, dynamic convolutional alignment, and DFL to maintain the detection accuracy in small targets and complex backgrounds.

**Figure 7:**
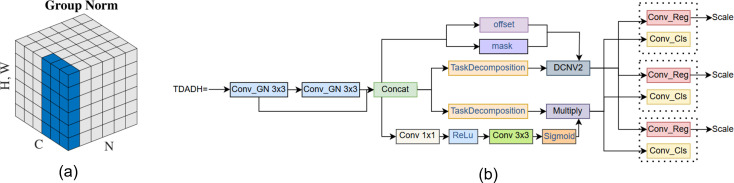
**(a)** GroupNorm Structure, **(b)** TDADH Detection Head Structure.

In the structure we introduce group normalization GroupNorm and shared convolution, GroupNorm has proven effective in FOCS (Tian et al., 2019) for enhancing localization and classification performance. The structure is shown in **Figure 7a**. TDADH detection head implements task dynamic alignment, uses the idea of task decomposition, makes full use of the advantages of GroupNorm and shared convolution, and is able to reduce the computational volume and complexity as much as possible to make the model lighter while maintaining the effective fusion of feature information. And the Scale module scales the features of different scales of different detection heads. The detection results from the multi-scale detection heads are fed into two shared 3x3 convolutions and the convolutions are spliced. Interaction features are generated after channel concatenation. Task decomposition is performed on the interaction features. The interaction features are used to generate mask and offset to enhance the interaction capability. offset offset and mask mask enable the convolution operation to dynamically adjust the positions of the sampling points and weight them according to the importance of the sampling points. This makes the convolution operation more flexible and powerful, significantly improving model performance, detection accuracy and robustness of feature extraction. Dynamic feature selection attention weights are generated using interaction features prior to task disassembly. The DyDCNV2 dynamic deformable convolution module spatially aligns the regression features to generate the category branches and regression branches. GroupNorm is shown in **Figure 4**, where N denotes the batch axis of the data, C is used as the channel axis, and H, W are used as the spatial axes.

In the TDADH detection header network structure shown in **Figure 7b**, the Conv_GN3×3 module represents the group normalized GroupNorm and the convolutional Conv, where 3×3 denotes that the convolutional kernel size is 3×3. The parts in front of the Scale module are shared weights except for Concat. The two blue group normalized convolution modules Conv_GN 3×3 share weights, the three blue prediction frame convolution modules Conv_Reg share weights, and the three red classification convolution modules Conv_Cls share weights. The Scale module is connected behind each Conv_Reg module, and the Scale module is used to scale the features at different scales.

## 3 Model training and result analysis

### 3.1 Experimental environment and configuration

The HPC platform used in this study is configured with an NVIDIA RTX 4090 GPU (24 GB VRAM) graphics card, using an Intel Xeon Platinum 8352V CPU (12 vCPUs, 2.10 GHz), and 90 GB RAM. the DLframeworks used are Python 3.10 and PyTorch 2.1.0, accelerated by CUDA with architecture version 12.1. The model was trained on the dataset by setting the image input size to 640×640 pixels, the initial learning rate to 0.01, the momentum set to 0.937, the batch size set to 16, the number of training rounds set to 400, and the stochastic gradient descent SGD optimizer was selected, and the number of data loading threads was 4. The selection of hyperparameters is based on a combination of prior experimental experience, general practices in the field, and preliminary experimental results on the dataset used in this study. These settings are also consistent with the configurations commonly used in YOLO-based architectures to ensure stable convergence and computational efficiency under current hardware conditions.

### 3.2 Evaluation metrics

In the task of maize seedling plant detection, accurately evaluating the model performance is crucial for the detection of maize seedling plants. The maize seedling detection process needs to consider both detection accuracy and speed. Lower computation, number of parameters, model size and inference time means that the model requires less computational resources to perform the task, can be more easily deployed to mobile terminals, and has better real-time processing performance. For model detection accuracy, this study chooses precision P (Precision), recall R (Recall) and mean average precision mAP (mean average precision) as the evaluation metrics. For model detection performance, model parameters, weights size after model training, FLOPs (Floating Point Operations per second) and FPS (Frames Per Second) are used as the evaluation indexes to measure the model’s computational complexity of the model.

Although the three metrics p, r, and ap are not direct in this paper, it helps to understand the other metrics. Precision: P reflects the ability of the model to correctly predict the samples of maize seedling class, which is calculated as shown in Equation 4.


(4) $$P=\frac{TP}{TP+FP}$$

(5) $$R=\frac{TP}{TP+FN}$$

Recall: R is the proportion of samples in the maize seedling category correctly predicted by the model to the total number of samples in the maize seedling category, which reflects the ability of the model to correctly identify all real instances of maize seedlings, calculated as shown in Equation 5.

where TP denotes the number of samples that are actually maize seedlings that are correctly predicted to be in that category. tN denotes the number of samples that are not actually maize seedlings that are correctly predicted to be in other categories; FP denotes the number of samples that are not actually maize seedlings that are incorrectly predicted to be in that category; and FN denotes the number of samples that are actually maize seedlings that are incorrectly predicted to be in other categories during the detection process. n denotes the number of categories In this study, only one category, corn seedling, is discussed, so at this time N = 1. AP denotes the area under the curve enclosed by precision-recall. mAP reflects the average identification precision of all the categories and is calculated as in Equation 6. mAP@0.5 indicates the average precision when the IoU threshold is 0.5.In this experiment, only one category of maize seedlings is discussed, so the AP value is the same as the mAP value. The mAP@0.5 is the average precision obtained when the intersection over union (IoU) threshold is set to 0.5.

The parameters are the total number of parameters to be trained in the network model, corresponding to the consumption of hardware memory resources, and are used to evaluate the spatial complexity of the model in M. The calculation formula is given by Equation 7:


(6) $$\text{m}AP=AP=\int_{0}^{1}\text{p}•r•dr$$

(7) $$P\text{ararmeter}=C_{\text{in}}\times C_{\text{out}}\times K^{2}+C_{\text{out}}$$

where, $C_{\text{in}}$ , $C_{\text{out}}$ are denoted as the number of input and output channels, respectively, and K are the width and height of the convolution kernel.

The computational amount is used to quantify the computational efficiency of the model, and a lower value indicates that the model requires less computational resources and is more efficient in performing the task. The computational formula is given by Equation 8 as follows:


(8) $$FLOP\text{s}=2\times (C_{\text{out}}\times K^{2}\times H_{\text{out}}\times W_{\text{out}}\times C_{\text{in}}+C_{\text{out}}$$

Where: $H_{\text{out}}$ , $W_{\text{out}}$ denotes the height and width of the output feature image; the factor 2 is because each multiplication and addition counts as 2 FLOPs.

FPS denotes the number of images detected by the model per second. Higher FPS value means better real time performance of the model. The formula is as follows (Equation 9).


(9) $$FPS=\frac{1}{T}$$

Where, T denotes the time taken by the model to detect each image in seconds. Note on FPS and hardware variability: The reported FPS values in this study were obtained using a high-end desktop platform configured with an NVIDIA RTX 4090 GPU (24 GB VRAM), Intel Xeon Platinum 8352V CPU, and 90 GB RAM. It should be noted that FPS is highly hardware-dependent and may vary significantly across devices. The proposed YOLOv8-FLY model is specifically designed to reduce parameter count, computational cost, and memory usage, making it suitable for deployment on lower-resource platforms such as UAV onboard systems or embedded edge devices. However, for accurate estimation of real-time performance in field applications, inference speed should be evaluated on the target deployment hardware.

### 3.3 Comparison experiments

In order to select the best model for the experiments, this study conducted comparison experiments on mainstream target detection models containing YOLOv3, YOLOv5s, YOLOv6, YOLOv8n, YOLOv8s, YOLOv8m, YOLOv8l, YOLOv8x, YOLOv9s, YOLOv10s, Faster R-CNN and SSDs. During the experiments, YOLOv8n with high detection accuracy and excellent lightweight performance was chosen as the benchmark model for the experiments. As shown in **Table 1**, YOLOv8n achieves a model performance of 96.4% mAP and 149.98 FPS with only 3.01 M number of parameters, 8.1 G of FLOPs of computation, and a weight file size of only 6.3 MB, which is significantly lower than the other models. Although some of the models in the table have higher mAP than the YOLOv8n model, all other aspects of the model performance are higher than the benchmark model, which is not conducive to the application of the model in lightweight scenarios such as embedded systems or real-time processing tasks.YOLOv8n has a much smaller model weight, number of parameters, and computation, as well as a high inference speed FPS. This balance between model accuracy, complexity, and resource utilization efficiency balance makes YOLOv8n the best choice for use in model lightweighting.

**Table 1:** Comparison of different model metrics.

Model	mAP50/%	Weights/MB	FLOPs/G	Parameters/M	FPS
YOLOv3	96.7	207.8	283.0	103.69	97.39
YOLOv5s	96.5	18.5	23.8	9.11	129.15
YOLOv6	96.3	8.7	11.8	4.23	147.99
YOLOv8n	96.4	6.3	8.1	3.01	149.98
YOLOv8s	96.5	22.5	28.4	11.13	130.92
YOLOv8m	96.4	52	78.7	25.84	134.4
YOLOv8l	96.6	87.6	164.8	43.61	97.01
YOLOv8x	96.6	136.7	257.4	68.12	92.64
YOLOv9s	96.3	15.2	26.7	7.17	37.13
YOLOv10s	96.6	16.5	21.4	7.22	119.23
Faster R-CNN	96.1	302.1	148.3	51.72	107.72
SSD	95.8	103.7	128.7	23.19	32.65
YOLOv8-FLY	96.5	3.5	7.4	1.58	146.3

**Figure 8:**
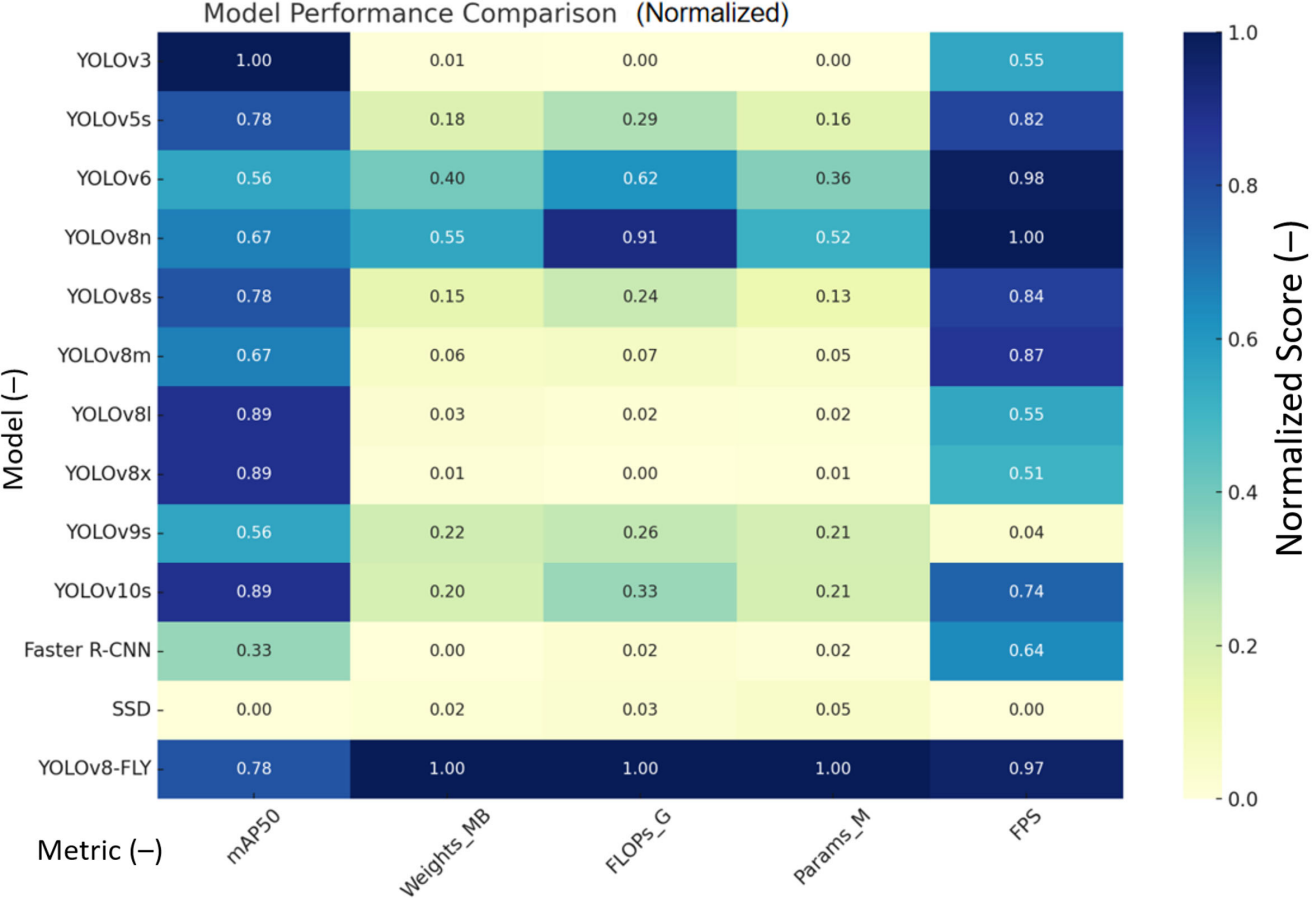
Normalized Heatmap of Metrics for Different Models.

The five performance metrics (mAP50, Weights, FLOPs, Params, FPS) in the above table are normalized to generate a comprehensive contrastive heat map. As shown in **Figure 8**: As can be seen from the figure: YOLOv8-FLY performs extremely well in almost all dimensions, achieving lightweight, fast, and high-precision. YOLOv8n, YOLOv6, YOLOv10s, etc. also achieve a good balance between speed and complexity; Faster R-CNN and SSD are relatively weak in terms of inference speed and model complexity.

**Figure 8** Note: 1. The darker the color (close to blue) in the heatmap, the better the performance; 2. For the ‘smaller is better’ metrics (e.g., Weights, FLOPs, Params), the inverse has been converted to unify the standard.

### 3.4 Ablation experiments

In order to better verify the optimization of the improved model relative to the original model, ablation experiments were conducted on seven combinations containing three network structures, all using the same maize seedling dataset, batch, and training cycle. The results of the ablation experiments are shown in **Table 2**. Line 1 in the table shows the original YOLOv8n model results, and √ indicates that the corresponding module was improved based on the YOLOv8n model. From the comparison in the table, it can be seen that the YOLOv8n model achieves the minimum model computation FLOPs of 6.9 G after adding the Rep_HGNetV2 module. This is because Rep_HGBlock, which is constructed by using the idea of reparameterisation, uses the multilayer lightweight convolution and efficient feature fusion strategy to enable the model to significantly reduce the model parameters, computation volume, and weight size. The model achieves the smallest model weight of 3.5MB with the addition of Rep_HGNetV2+TDADH module, and the reduction of inference speed FPS is more obvious at 54.57. The model parameters of the three related comparison experiments in the table that include the TDADH detection header are smaller than the other comparison experiments, which proves the effectiveness of the shared convolution and task alignment strategy to reduce the number of model parameters in the TDADH detection header. alignment strategy to reduce the number of model parameters is effective. The YOLOv8n-FLY model combining all the improved structures achieves 96.5% accuracy, 3.5 MB of model weights, 7.4 G of computational FLOPs, 1.58 M of model parameter counts, and 146.3 FPS. the model maintains high detection accuracy while achieving smaller model weights and parameter counts, lower computational effort, and higher real-time inference speeds to It meets the demand for efficient, lightweight and convenient deployment of maize seedling detection in the field.

**Table 2:** Comparison of metrics in ablation experiments.

number	Rep_HGNetV2	Bifpn	TDADH	mAP50/%	Weights/MB	FLOPs/G	Parameters/M	FPS
1				96.4	6.3	8.1	3.01	149.98
2	✓			96.4	5.0	6.9	2.34	123.97
3		✓		96.1	6.3	8.2	3.02	129.12
4			✓	96.3	4.7	8.6	2.24	59.68
5	✓	✓		96.6	5.1	7.0	2.38	142.07
6	✓		✓	96.5	3.5	7.4	1.68	54.57
7		✓	✓	96.3	4.7	8.6	2.24	34.65
8	✓	✓	✓	96.5	3.5	7.4	1.58	146.3

**Figure 9:**
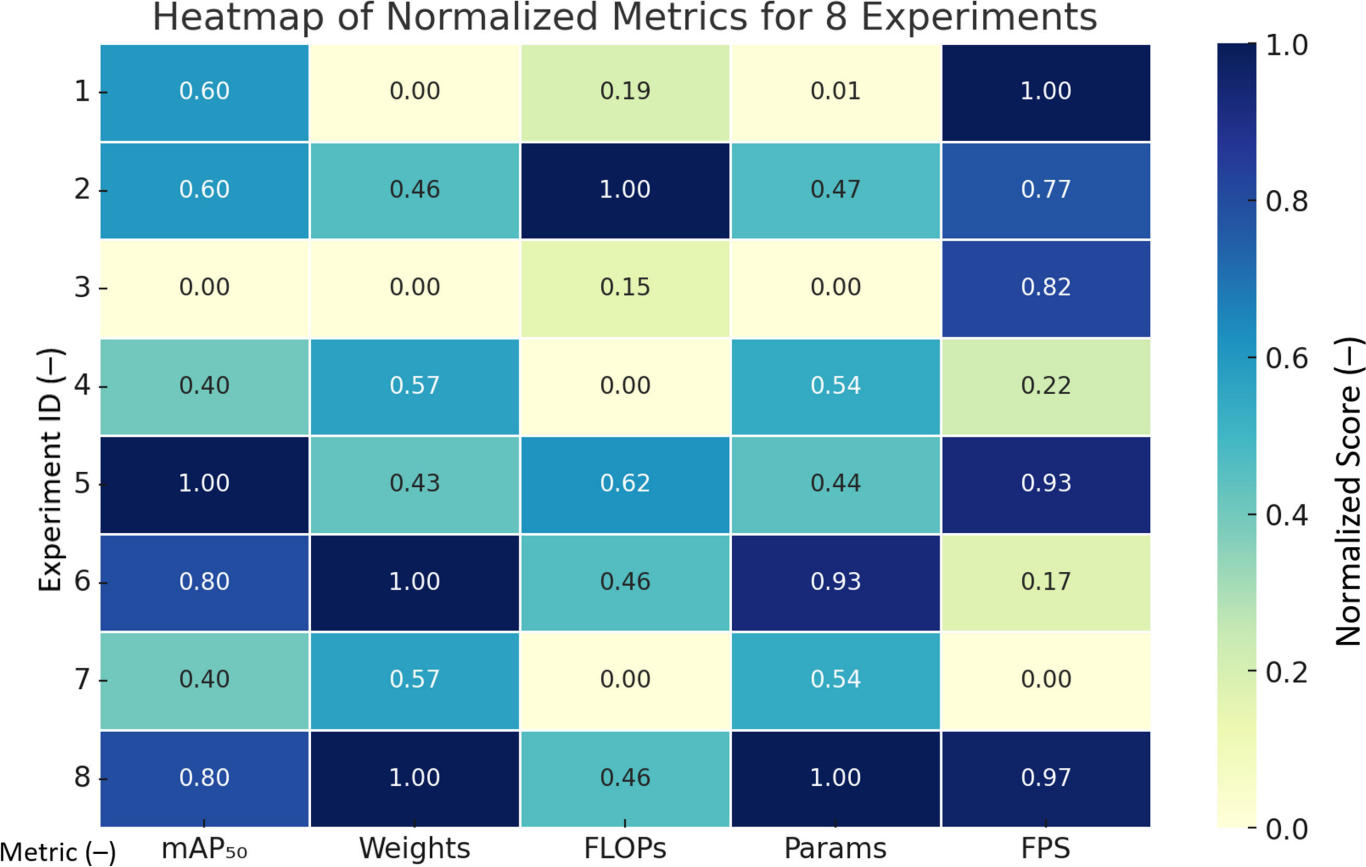
Normalized Heatmap of Metrics for Ablation Experiments.

The normalized performance of the above ablation experiments on the five metrics (the closer the value is to 1, the better it is) is generated into a heat map as shown in **Figure 9** below, which shows that the overall performance of the YOLOv8-FLY model is very well balanced, with almost all dimensions close to 1. The original model is the strongest in terms of FPS (1.00), but poorer in terms of model complexity; the performance of other metrics of the model have decreased to varying degrees.

**Figure 9** Note: 1. Rows indicate experiment numbers 1 - 8; 2. Columns are the names of the metrics that have been normalized; 3. The higher the accuracy mAP_50_, the better; 4. The higher the complexity of the model that has been back-normalized in terms of Weights, FLOPs, and Params, the better; 5. The speed of detection FPS The higher the better; 6. Color shades indicate the relative goodness of the metric.

### 3.5 Detection head comparison

During the experiments, the effects of different detection heads on improving the relevant parameters of the model were verified and compared. In order to fully assess the performance of TDADH in the YOLOv8n model, TDADH was analyzed in a comparative experiment with other commonly used small target detection heads. The aim was to assess the differences in lightweight and real-time detection performance of different detection heads in the task of detecting maize seedling targets. The preconditions, backbone network and neck network of the comparison model were consistent, and the p2 layer of the detection head was added in Smallhead, and the normal detection head was replaced with the dynamic detection head in Dyhead. The performance metrics of different detection heads are demonstrated as shown in **Table 3**. From the table, it can be compared that the detection accuracy of the model remains basically the same when replacing different detection heads. The performance of adding p2 detection head and dynamic detection head Dyhead are not as good as the performance of the improved detection head TDADH in terms of lightweighting and inference speed.

**Table 3:** Comparison of metrics for different detection head experiments.

Model	mAP50/%	Weights/MB	FLOPs/G	Parameters/M	FPS
Detect	96.6	5.1	7.0	2.38	142.07
TDADH	96.5	3.5	7.4	1.58	146.3
Smallhead	96.6	5.0	11	2.26	115.02
Dyhead	96.6	6.7	12.7	3.18	33.03

**Figure 10:**
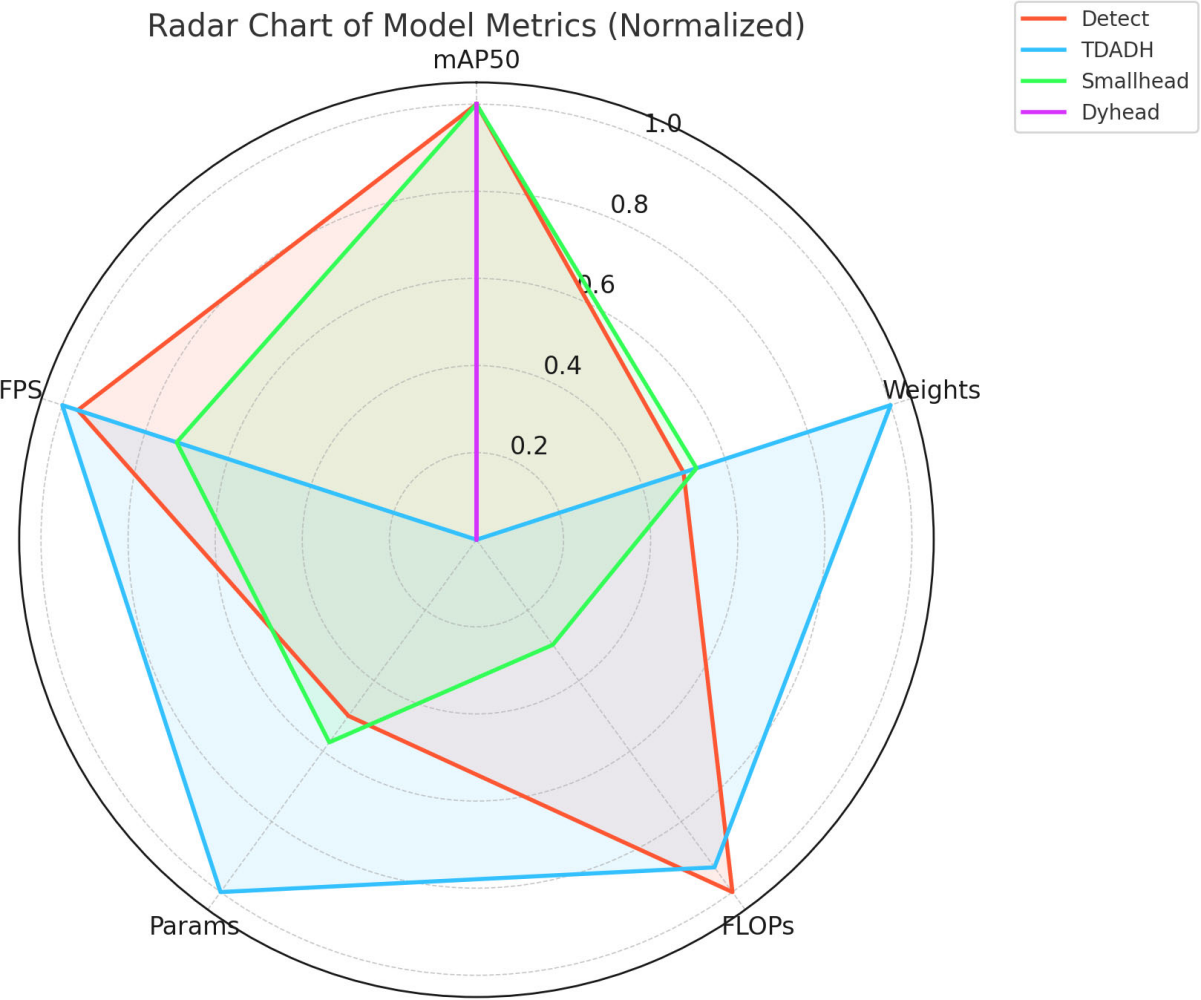
Normalized Radar Chart of Metrics for Different Detection Heads.

A comprehensive analysis of the normalized performance of each detector head on the five metrics generates radar plots as in **Figure 10**. The graph of TDADH approximates a pentagon, indicating that it has a balanced or even superior performance in all the metrics, especially in terms of parameter, model size, and FPS, making it the lightest and most efficient model; Detect also has a larger area and is only marginally inferior in terms of parameter and model size; and Dyhead sacrifices resource for accuracy, excelling only in mAP50, with severe graph shrinkage in the remaining dimensions; Smallhead is average overall.

**Figure 10** Note: The larger the graph area, the better the overall performance.

### 3.6 Experimental results and analysis

**Figure 11:**
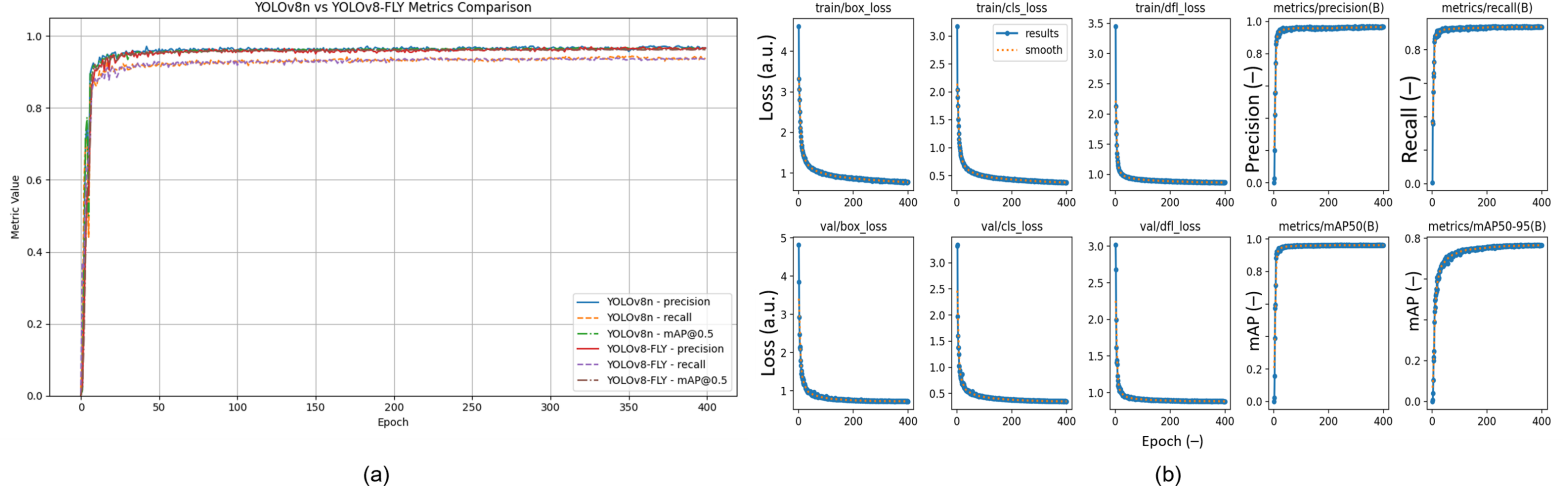
**(a)** Comparison of Precision, Recall, and mAP50 Curves between YOLOv8n and YOLOv8-FLY, **(b)** Training and validation performance curves of the YOLOv8-FLY model over 400 epochs. The loss functions include Box Loss, Classification Loss, and DFL for both training and validation sets. Performance metrics include P, R, mAP@50, and mAP@50–95.

In order to verify the effectiveness of the improved lightweight model in this study, a comparison experiment between the pre-improved YOLOv8n and the lightweight YOLOv8-FLY model was conducted in the maize seedling detection task. The experimental detection metrics are shown in **Figure 11** as the trends of the three metrics of the model, accuracy P, recall R, and mean average precision mAP50, during the training cycle. From the curves in the figure, it can be seen that both models converge and stabilize faster during the training process, and lightweighting does not bring training instability or serious precision loss. Among them, YOLOv8-FLY is basically the same as YOLOv8n in terms of accuracy performance, and even slightly improves in some phases, which indicates that the lightweight model does not bring obvious performance loss, and has good stability and generalization ability.

**Figure 12:**
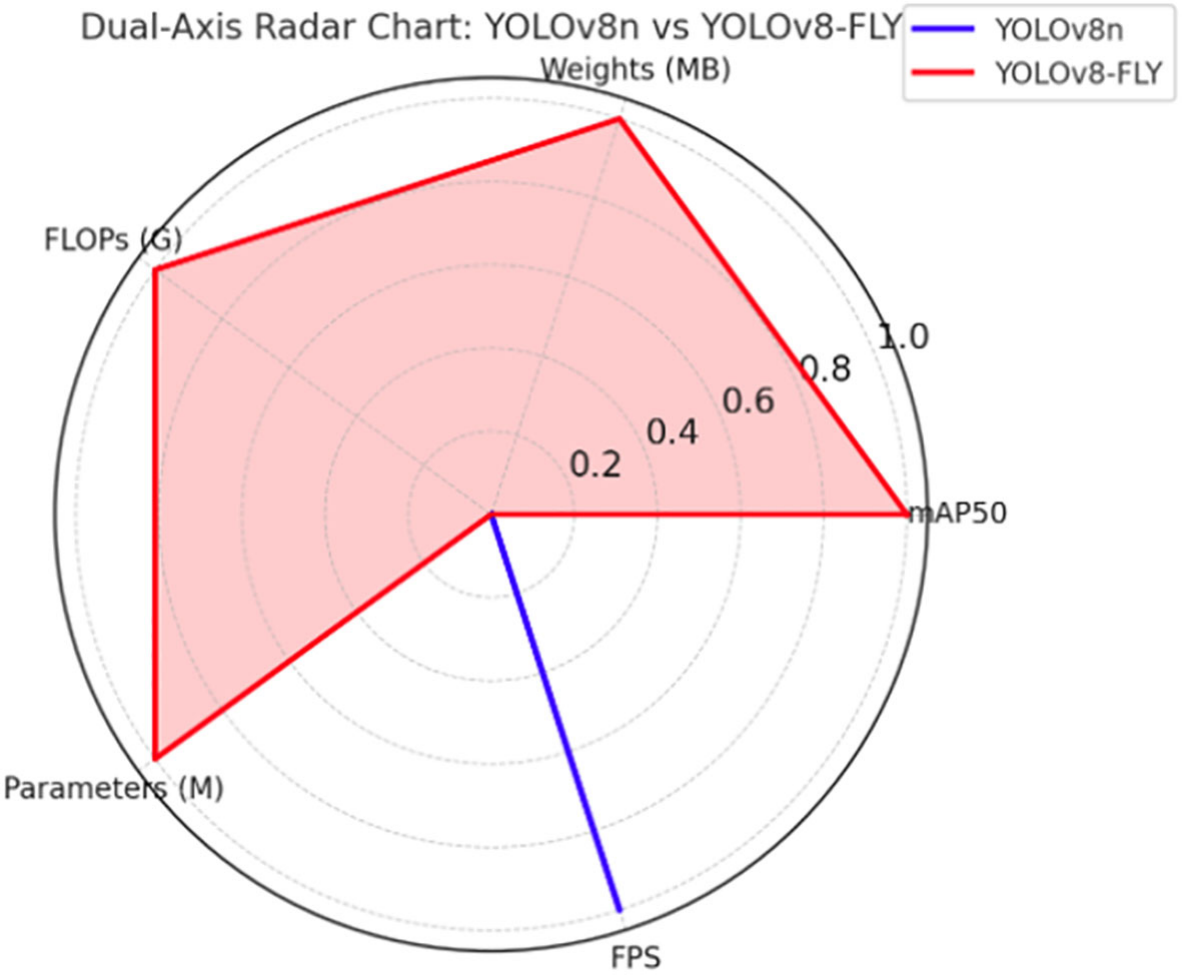
Normalized Biaxial Radar Chart of Five-Dimensional Performance for YOLOv8n and YOLOv8-FLY Models.

On the validation set, the YOLOv8-FLY model maintains a 96.5% mAP comparable to the original YOLOv8n model, and achieves significant optimization in terms of model complexity. Specifically, the YOLOv8-FLY model achieves significant reductions in weight size, computational effort, and number of parameters. Although the inference speed of YOLOv8-FLY is almost equal to that of YOLOv8n (only a reduction of 3.68 FPS), it still maintains an extremely high real-time detection capability. The normalized biaxial radar chart of the model metrics before and after the improvement is shown in **Figure 12**. Therefore, YOLOv8-FLY significantly reduces the model complexity and deployment resource consumption while ensuring the detection accuracy, and is suitable for deployment in resource-constrained edge devices, which has a better application prospect in real field scenarios.

**Figure 12** Note: In the biaxial radar chart, the forward axis is used for accuracy-related metrics and the reverse axis is used for complexity-related metrics. When normalized, mAP50 and FPS are forward metrics, the bigger the better; while Weights, FLOPs and Parameters are reverse metrics, the smaller the lighter the weight when inverted during normalization. The more the polygon boundaries in the figure extend outwards, the better the model performs on the corresponding indicators. From the figure, it can be clearly seen that YOLOv8-FLY significantly outperforms YOLOv8n in the dimensions of Weights, Parameters and FLOPs, forming an ‘outwardly expanding’ pentagon, while keeping the mAP and FPS levels almost unchanged.

### 3.7 Heat map analysis

**Figure 13:**
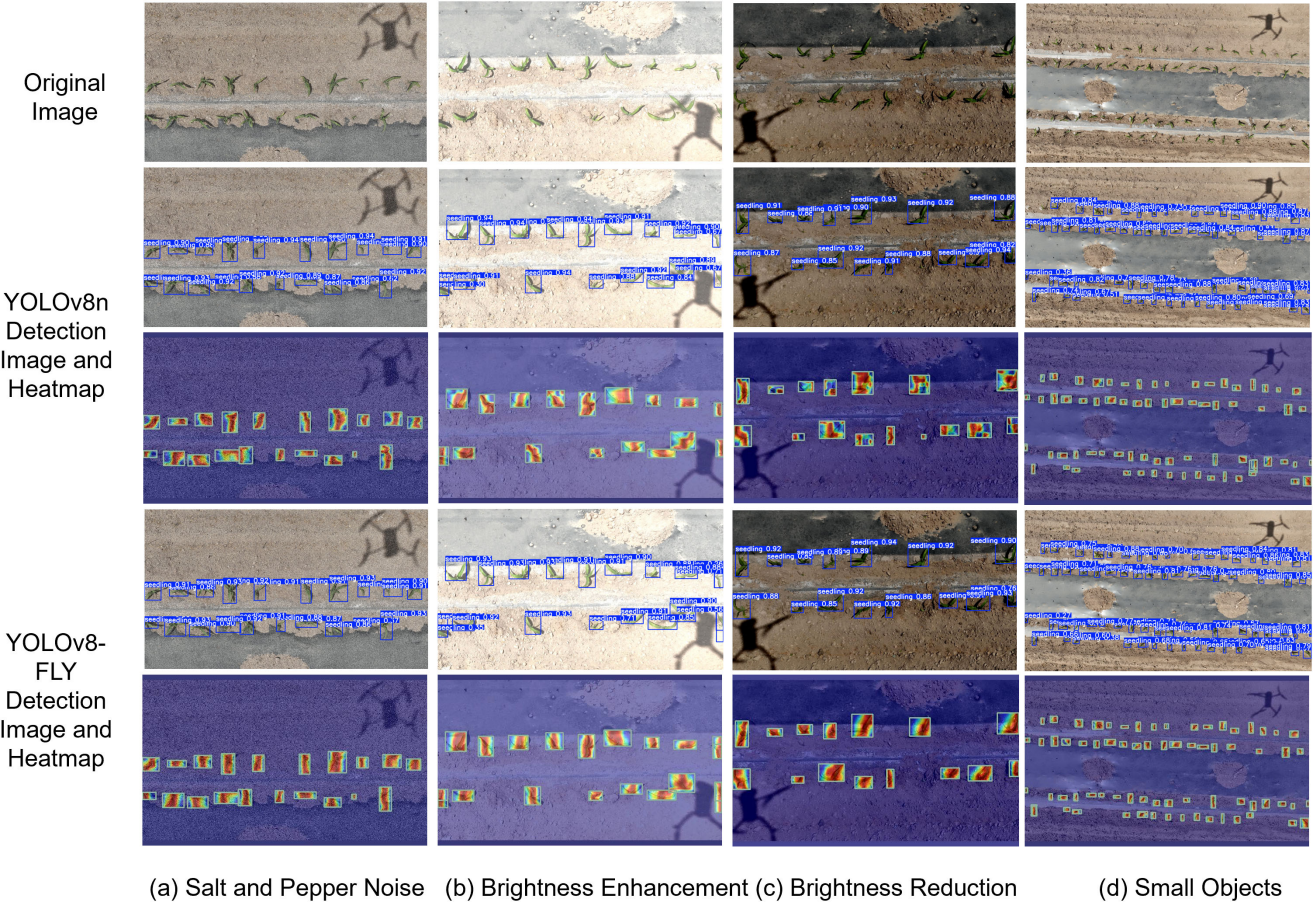
Comparative Analysis of Model Detection Heatmaps Before and After Improvement. **(a)** Salt and Pepper Noise,**(b)** Brightness Enhancement, **(c)** Brightness Reduction, **(d)** Small Objects.

In order to more clearly demonstrate the feature extraction ability of the model and the interpretability of the network, this study uses the Grad-CAM++ visualization of CNNs to visualize the features of the YOLOv8n model before and after the improvement, as shown in **Figure 13**. Grad-CAM++ is an improvement of Grad-CAM (Selvaraju et al., 2017), which is able to more accurately and meticulously visualize the CNN image regions of interest when making classification decisions. By introducing a finer-grained weighting algorithm, multiple image regions that contribute to classification can be better captured. Using higher-order derivatives (second-order and third-order gradients) to compute the weights provides a more accurate measure of the contribution of the feature map and generates a more detailed and accurate localization heatmap. Grad-CAM++ provides a significant improvement in the ability to recognize small targets and localize targets at a fine-grained level.

Red and yellow colors in the heat map indicate high attention areas that contribute more to the prediction results of the model. Blue and green indicate low attention areas that contribute less to the prediction results of the model. The heat map shows that the red area of the improved YOLOv8-FLY model is larger than that of the original model, which indicates that the model exchanges contextual semantic information more adequately, and is more accurate and comprehensive for multi-scale feature fusion. The brightness of the contour region of the improved model target is higher compared to the original model, indicating that the improved model feature fusion is more comprehensive attention focusing on a larger range, the location of the thermal region overlaps more with the target object, and the model decision is clearer and more credible; the original model feature fusion ability is lower, and the range of the highlighted region covering the object in the detection frame is smaller; **Figure 13c** shows that the original model thermal map produces misdetection of the background. **Figure 13d** the improved model has better perception of small target detection ability. Comparing the heat maps of YOLOv8n and the improved YOLOv8-FLY model, it can be seen that the improved YOLOv8-FLY model is able to capture rich and comprehensive features with better robustness and adaptability when dealing with weak semantic target information in complex scenes.

### 3.8 Maize seedling plant detection system design

**Figure 14:**
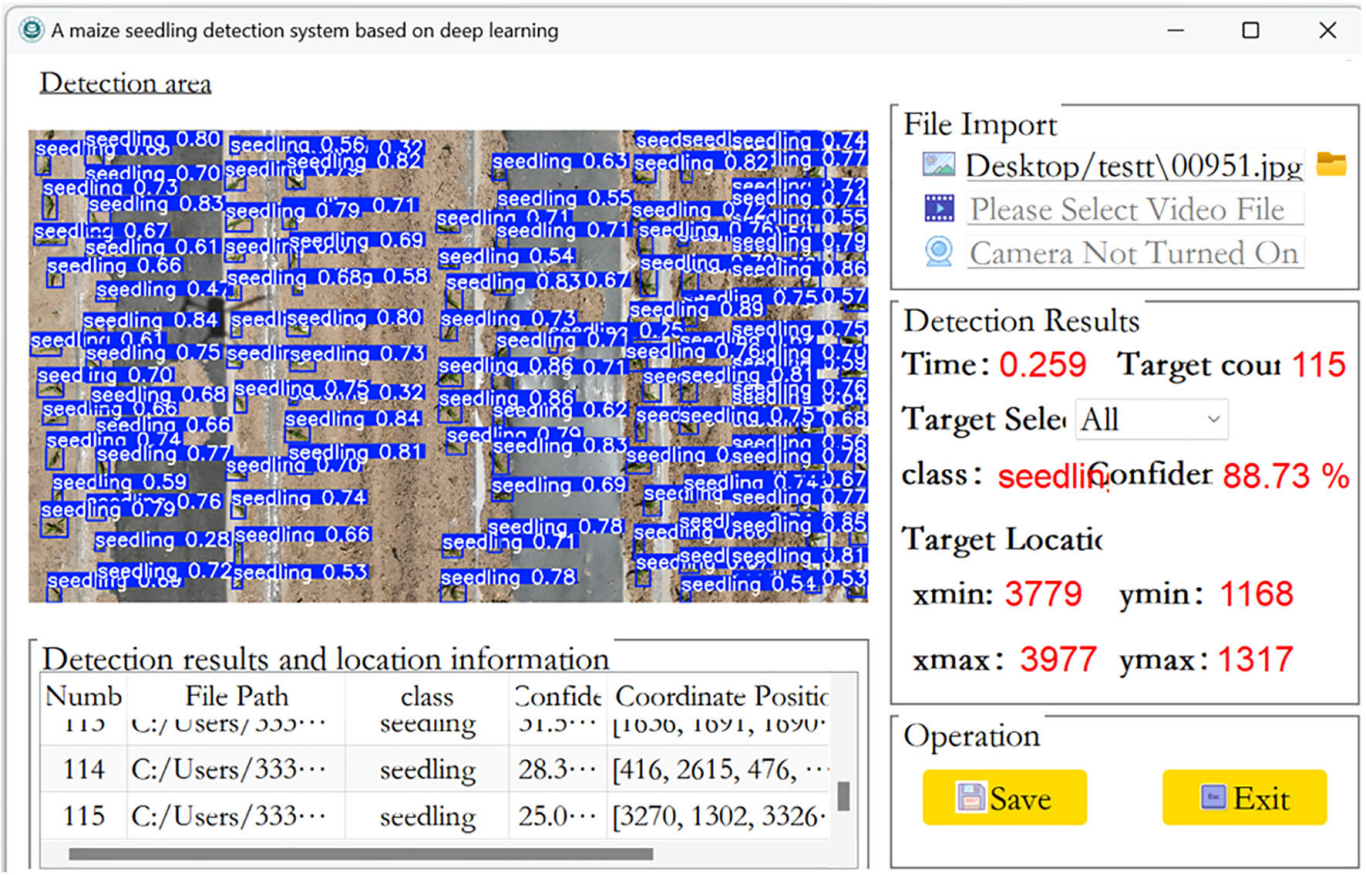
Interface of the Maize Seedling Detection System.

Pyqt5 is a GUI (Graphical User Interface) programming framework for the Python language. It is developed based on the Qt library, combining the rich functionality of Qt with the simplicity and ease of use of Python. Developers can easily create various interactive application interfaces using Pyqt5. In this study, a real-time maize seedling plant detection system based on PyQt5 was designed. The system interface is shown in **Figure 14**. The system has several important features. Firstly, it allows the user to select pre-trained maize seedling detection models to provide a strong technical basis for subsequent detection. Second, the system supports the detection of corn seedling images and video files collected in the field, and the system quickly and accurately displays images of detected corn seedling plants and their locations. It also displays the number of seedling plant targets detected and the number of frames that can be processed per second. These data are important for evaluating detection efficiency and effectiveness. Detection results and location information The detection of images under the target folder generates a list of detected maize seedling plants, the target category, the confidence level of the detected target and the coordinate location of the target in the image. In addition, the system supports real-time maize seedling detection in conjunction with a UAV camera.

## 4 Discussion

In complex field environments, maize seedlings present significant challenges for object detection due to their small size, high morphological variability, and frequent occlusions. To address this, we propose YOLOv8-FLY, a lightweight maize seedling detection model tailored for drone-based RGB imagery. This model achieves high detection accuracy while significantly reducing computational complexity and parameter count, demonstrating excellent performance and practical application potential.

Compared to mainstream lightweight models, YOLOv8-FLY achieves a better balance between accuracy, speed, and model size. On our self-built maize seedling dataset, YOLOv8-FLY achieves a mAP of 96.5%, with 1.58 M parameters, a model size of just 3.5 MB, and an inference speed of up to 146.3 FPS. This result outperforms the lightweight maize seedling counting model proposed by Gao et al. (2022), which was built based on an improved YOLOv4 (mAP 97.03%, model size 71.69 MB, model parameters 18.793 M, FPS 22.92), significantly reducing model size and improving real-time performance while maintaining high accuracy.

In recent years, researchers have also explored various lightweight optimizations of the YOLO framework. Zhao et al. (2023) introduced BiFPN into YOLOv7 to propose the LW-YOLOv7 model, achieving 93.2% mAP and 90 FPS, but with a parameter count of 59.4 M, resulting in high deployment costs. In contrast, this study employs BiFPN to achieve more efficient multi-scale feature fusion, enhancing robustness in UAV images of farmland with frequent occlusions and lighting changes. Li et al. (2025) proposed PSDS-YOLOv8, which achieved 96.5% mAP in wheat ear detection, but its parameter count was 6.8 M, exceeding YOLOv8-FLY’s 1.58 M, indicating that our model offers a significant lightweight advantage while maintaining accuracy. To further enhance lightweight feature extraction capabilities, we introduce the Rep_HGNetV2 backbone network and design the TDADH detection head, significantly reducing redundant parameters in the detection phase. These structural innovations provide valuable insights for the application of small object detection in precision agriculture.

Unlike most methods that are only evaluated under experimental conditions, this study integrates YOLOv8-FLY into a complete detection and counting system. This system can run in real time on a standard lightweight laptop, enabling efficient detection and counting of UAV images or real-time video streams, and displaying key information such as frame rate, category, confidence level, and bounding box coordinates in real time via a graphical interface. This deployability is crucial for field applications such as seedling counting and replanting decisions. Similar deployment capabilities are also demonstrated in the YOLOv8s-Longan model proposed by J. Li et al. (2025) and the S-YOLO model proposed by Sun (2024), but this model outperforms them in terms of inference speed and model compression.

However, the model still has limitations: detection accuracy may decrease under extreme conditions (such as strong shadows, soil compaction, and weed obstruction); despite the use of data augmentation, it is still difficult to cover rare or abnormal scenarios; the current dataset lacks cross-regional and cross-seasonal diversity, and the model’s generalization ability still needs further verification.

Future work should explore the construction of cross-regional and cross-seasonal multi-source datasets to enhance the model’s robustness and generalization ability; attempt to integrate multi-spectral or depth information to enhance feature expression capabilities in complex environments; and design systematic ablation experiments to deeply explore the relationship between data augmentation strategies and model performance, avoiding overfitting and sample redundancy.

In summary, YOLOv8-FLY has achieved significant improvements in real-time maize seedling detection and laid the foundation for research on lightweight, deployable precision agriculture models. Through systematic comparisons with existing research and detailed analyses of structural innovations, this study provides a feasible and reliable solution for small-object crop detection in agricultural scenarios.

## 5 Conclusion

This study proposes a lightweight, high-performance object detection model, YOLOv8-FLY, specifically designed for detecting maize seedlings in RGB images captured by drones under water-fertilizer integration conditions. Addressing challenges such as small target sizes, severe occlusions, and complex field backgrounds, this model integrates Rep_HGNetV2, BiFPN, and TDADH detection heads, achieving a highly competitive balance between detection accuracy, model size, and inference speed.

Experimental results significantly outperform multiple mainstream lightweight detectors, demonstrating immense potential for real-time, field-level deployment in smart agriculture scenarios. YOLOv8-FLY has been validated in actual detection systems, supporting real-time visualization and decision support, marking a key step towards field-ready smart agriculture solutions. In addition to its performance advantages, the model offers a scalable, flexible framework that can be further adapted to other crop types or environmental conditions. However, its performance remains limited under extreme field conditions.

Future work will focus on enhancing the model’s generalization capability and robustness through multi-modal image fusion and optimized deployment on edge computing platforms such as Jetson Nano or Xavier NX. Additionally, current experiments are limited to static images, and future research will extend YOLOv8-FLY to video-based monitoring and multi-angle drone imagery to enrich spatio-temporal perception capabilities, supporting continuous field monitoring and dynamic decision-making. In conclusion, YOLOv8-FLY provides a compact, efficient, and deployable solution for drone-based crop monitoring, laying a solid foundation for scalable, real-time applications in precision agriculture.

## Data Availability

The original contributions presented in the study are included in the article/supplementary material. Further inquiries can be directed to the corresponding author/s.
